# An experimental test of the growth rate hypothesis as a predictive framework for microevolutionary adaptation

**DOI:** 10.1002/ecy.3853

**Published:** 2022-10-23

**Authors:** Kimberley D. Lemmen, Libin Zhou, Spiros Papakostas, Steven A. J. Declerck

**Affiliations:** ^1^ Department of Aquatic Ecology Netherlands Institute of Ecology (NIOO‐KNAW) Wageningen Netherlands; ^2^ Department of Biology University of Turku Turku Finland; ^3^ Department of Biology, Laboratory of Aquatic Ecology, Evolution and Conservation KU Leuven Leuven Belgium; ^4^ Present address: Department of Evolutionary Biology and Environmental Studies University of Zurich Zurich Switzerland; ^5^ Present address: Institute of Ecology, College of Urban and Environmental Science, and Key Laboratory for Earth Surface Processes of the Ministry of Education Peking University Beijing China; ^6^ Present address: Department of Genetics, Development and Molecular Biology School of Biology, Aristotle University of Thessaloniki Thessaloniki Greece

**Keywords:** *Brachionus calyciflorus*, contemporary evolution, ecological stoichiometry, experimental evolution, intraspecific genetic variation, phosphorus limitation, rapid adaptation, rotifera, zooplankton

## Abstract

The growth rate hypothesis (GRH) posits that the relative body phosphorus content of an organism is positively related to somatic growth rate, as protein synthesis, which is necessary for growth, requires P‐rich rRNA. This hypothesis has strong support at the interspecific level. Here, we explore the use of the GRH to predict microevolutionary responses in consumer body stoichiometry. For this, we subjected populations of the rotifer *Brachionus calyciflorus* to selection for fast population growth rate (PGR) in P‐rich (HPF) and P‐poor (LPF) food environments. With common garden transplant experiments, we demonstrate that in HP populations evolution toward increased PGR was concomitant with an increase in relative phosphorus content. In contrast, LP populations evolved higher PGR without an increase in relative phosphorus content. We conclude that the GRH has the potential to predict microevolutionary change, but that its application is contingent on the environmental context. Our results highlight the potential of cryptic evolution in determining the performance response of populations to elemental limitation of their food resources.

## INTRODUCTION

Microevolutionary responses to environmental change frequently occur on ecologically relevant timescales (Hairston Jr. et al., 2005; Hendry, 2016) and have the potential to be predictable at the phenotypic level (Lässig et al., 2017). Given the unprecedented rate and magnitude of contemporary environmental change (Collins et al., 2013; Elser et al., 2009), understanding the trajectory of adaptive responses within populations becomes increasingly relevant. Ecological stoichiometry has utilized the flow of elements and their relative abundances to better understand ecological patterns and processes (Sterner & Elser, 2002). This framework could also be useful to predict evolutionary responses as the quantity and ratios of elements can act both as a selective pressure (e.g., resource quality) or as features that respond to selection (e.g., elemental composition) (Leal et al., 2017; Matthews et al., 2011). Considering selective forces in a stoichiometric context may, therefore, allow us to forecast evolutionary trajectories and their ecological impacts.

A key concept in ecological stoichiometry is the growth rate hypothesis (GRH). The GRH posits that the relative abundance of phosphorus in organisms (often quantified as the somatic phosphorus: carbon ratio, or P:C) is positively related to somatic growth rate, because growth requires protein synthesis, which depends on P‐rich ribosomes (Elser et al., 1996). In organisms where nucleic acids represent a large proportion of somatic P, such as small‐bodied invertebrates (Gillooly et al., 2005; Vrede et al., 1999), changes in the abundance of ribosomal RNA (rRNA), can affect whole‐body stoichiometry. In such organisms the GRH posits that differences in somatic P:C can be attributed to differential investment in protein synthesis (Sterner & Elser, 2002). Multiple studies provide empirical support for the GRH via correlations between SGR and RNA or P:C ratios across taxa (Elser et al., 1996, 2000, 2003; Ferrão‐Filho et al., 2007; Mouginot et al., 2014).

The GRH holds great potential as a framework for predicting microevolutionary change in populations. For example, knowledge of intraspecific genetic variation for relative somatic P‐content would allow us to predict the capacity of populations to evolve faster growth rates. Alternatively, it could be used to predict changes in somatic P in response to environmental contexts that select for fast population growth. For the GRH to be used as a predictive microevolutionary framework, organismal P:C and somatic growth rate both need to be heritable and show a strong genetic correlation. Currently, there is insufficient evidence of such correlation. Many of the studies investigating the GRH at the intraspecific level have reported a strong physiological association between P:C and somatic growth rate. However, because most of these studies have been conducted with single genotypes, they do not provide evidence for a genetic association between these traits (e.g., Acharya et al., 2004; DeMott et al., 1998; Kyle et al., 2006). Studies using multiple genotypes rarely quantify the genetic component of the response (e.g., Fink & Von Elert, 2006; González et al., 2014; Prater et al., 2018). The limited number of studies that do allow for a direct test of GRH predictions at the genotype level have reported evidence for considerable heritable variation in SGR but less so for P:C, and were inconclusive regarding the genetic relationship between both variables (Arnold et al., 2004; Liess et al., 2013; Sherman et al., 2017; Weider et al., 2004).

Here, we applied an experimental evolution approach to evaluate the power of the GRH to predict evolutionary responses of populations to selection for fast population growth rate (PGR). According to the GRH an increased investment in protein synthesis to achieve higher growth rates should result in an elevated somatic P:C ratio. When applied in a microevolutionary context, and assuming that SGR and PGR are strongly associated (Lampert & Trubetskova, 1996; Zhou & Declerck, 2019), we expect that populations evolving in response to selection for fast population growth should become dominated by fast growing genotypes with relatively high somatic P:C (Gorokhova et al., 2002). Alternatively, populations may be able to respond to selection for fast population growth without a simultaneous increase in P‐content (e.g., through a more efficient P‐metabolism or altered life history). As such, we anticipate the power of the GRH to predict evolutionary responses will be influenced by environmental P‐availability as reliance on P‐rich protein synthesis should be especially maladaptive in a P‐poor environment (Seidendorf et al., 2010; Sterner & Elser, 2002; Sterner & Hessen, 1994). To test these predictions, we subjected genetically diverse populations of the microzooplankton *Brachionus calyciflorus* to a culturing regime selecting for fast population growth using food at satiating concentrations that was either P‐rich or P‐poor. We then evaluated evolutionary responses by rearing the evolved and ancestral populations in a common garden experiment to compare population‐level traits associated with fitness and P‐stoichiometry. Our study is unique in that it empirically tests the idea that evolution toward increased population growth rate will be accompanied by an increase in body P‐content. Therefore, it allows us to explore the applicability of the GRH at the intraspecific level, and to test its ability to predict microevolutionary trajectories.

## MATERIALS AND METHODS

### Model organism

*Brachionus calyciflorus*, is a cyclical parthenogenetic planktonic monogonont rotifer, capable of reproducing asexually and sexually. Asexual reproduction produces subitaneous eggs allowing for rapid clonal population growth. In contrast, sexual reproduction produces diapausing embryos in so‐called “resting eggs” (Stelzer, 2017). The shift from asexual to sexual reproduction in rotifers is triggered by a change in the environmental conditions (Schröder, 2005) such as temperature and photoperiod (Gilbert, 2020; Pourriot & Snell, 1983). In *Brachionus* the best‐studied environmental cue is high population density of conspecifics (Stelzer & Snell, 2003), although other conditions such as food quality (Aránguiz‐acuña and Ramos‐Jiliberto Aránguiz‐Acuña & Ramos‐Jiliberto, 2014) and age (Fussmann et al., 2007; Schröder & Gilbert, 2004) can influence the reproduction mode of maternal individuals as well. The propensity for sex varies between genotypes (Becks & Agrawal, 2013), and high rates of sex typically result in reduced PGR (Serra & Snell, 2009; Stelzer, 2011).

### Origin and maintenance of algal and rotifer cultures

We used 30 distinct genotypes to initiate the evolution experiment (further referred to as “seed” genotypes; Appendix S1: Table S1). *B. calyciflorus* is part of a cryptic species complex which hitherto is comprised of four cryptic species (Michaloudi et al., 2018) that often hybridize (Papakostas et al., 2016). Our microsatellite analysis showed evidence of hybridization between the sister species *B. calyciflorus* and *B. elevatus* for seven of the “seed” genotypes (Appendix S2). We included these genotypes to incorporate genetic diversity representative of many natural populations. All genotypes were maintained in asexually reproducing stock cultures with nutrient replete resources (Appendix S2).

We used the motile green algae *Chlamydomonas reinhardtii* as a food resource in all experiments. To produce P‐rich (“HPF”: molar C:P ratio 121 ± 11.9 SE) and P‐poor (“LPF”: molar C:P 671 ± 9.9 SE) algae we varied the P‐content of the COMBO medium (Kilham et al., 1998) and light intensity (Appendix S2).

### Evolution experiment

To initiate the evolution experiment we assembled 14 replicate populations with identical genetic composition by combining two females with a single asexual egg from each of the seed genotypes. We randomly allocated seven of the populations to a P‐rich (HPF) and the other seven populations to a P‐poor (LPF) diet. All populations were cultured in 48 ml of the designated algal suspension at a concentration of 1550 μmol L^−1^ C and maintained in the dark at a constant temperature of 24 ± 1°C.

Every 24 h we transferred 60 haphazardly selected individuals and all resting eggs from each population to a new culturing flask with a fresh food suspension. By transferring a subset of the populations daily, we selected for fast clonal population growth as genotypes that produced the most offspring were more likely to be transferred. Food concentrations were provided ad libitum, which prevented density regulation of PGR due to intraspecific exploitative competition (i.e., density dependence). By transferring diapausing eggs, we allowed for sexually recombinant genotypes to establish.

After the daily transfer, we counted the remaining individuals. We calculated PGR as (ln*N*_*t*_ − ln*N*_0_)/*t*, where *N*_0_ and *N*_*t*_ represent the population size at the start and end of each 24‐h period, and *t* the duration of the period in days. The evolution experiment lasted 36 days. At the end of the experiment, we performed a microsatellite analysis to determine the genetic composition of each final population (Appendix S2). Following the conclusion of the experiment all populations were maintained in the culturing conditions of the evolution experiment for later use in common garden experiments.

### Common garden 1 (CG1): PGR and fraction of sexual individuals

Using the evolved populations, we performed a common garden experiment to test for genetic adaptation to selection for fast growth in the two food quality treatments. Due to logistical constraints, we randomly chose five of the seven evolved populations per selection treatment (Appendix S1: Table S2). We cultured four technical replicates of each of these populations in each common garden environment (HPF or LPF). In addition, to estimate the ancestral state we cultured one population for each of 10 randomly selected seed genotypes in each common garden environment.

We initiated each experimental unit with 10 rotifers and provided 8 ml of algal suspension at a concentration of 1550 μmol L^−1^ C. The first common garden lasted for 15 days. Every 24 h we transferred 10 haphazardly chosen individuals from each experimental unit into a fresh algal suspension, resting eggs were not transferred. We counted the remaining animals to estimate PGR and preserved them in 4% formalin solution. Data from the first 5 days were omitted from the calculations to avoid maternal effects from previous culturing conditions (Zhou & Declerck, 2020). To determine the fraction of sexual females for each replicate, we examined all preserved individuals and determined the number and type of eggs they carried (Appendix S2). The fraction of sexual females was defined as the number of females with sexual eggs (male and diapausing eggs) divided by the total number of egg‐bearing individuals (adults with male, diapausing, or amictic eggs).

### Common garden 2 (CG2): Rotifer elemental composition

A second common garden experiment was performed to evaluate the effect of selection history on organismal carbon (C), nitrogen (N), and phosphorus (P) content. The design of this experiment was similar to CG1; however, as quantifying rotifer elemental body composition requires a large number of individuals in the same body condition (i.e., age and reproductive stage), we applied an upscaled culturing method for CG2 (Appendix S2). The microsatellite analysis revealed some populations were dominated by the same genotype, we removed two populations from the experimental design to avoid redundancy (Appendix S1: Table S2).

### Life history experiment

As propensity for sex is known to strongly affect PGR (Stelzer, 2011), we conducted an abbreviated life table to assess the proportion of sexual individuals in LP‐ and HP‐evolved populations in LPF diet (see details in Appendix S2).

### Data analysis

To evaluate temporal trends in PGR during the evolution experiment, we compared the fit of two alternative models, a piecewise and linear regression model (Appendix S3). As the rate at which de novo genetic variation is generated may impact the pace of the adaptive response of a population to a given selection regime, we tested if resource quality (i.e., HPF, LPF) determined the total number of recombinant genotypes that potentially may have entered the populations of the selection experiment. To do so, we compared the total number of (sexually produced) resting eggs produced by the experimental populations in both food quality treatments using a linear model.

Microsatellite analysis revealed the existence of two different types of populations (Appendix S1: Table S2): (i) populations composed of one of two of the original seed clones identified as hybrids (further referred to as “hybrid” populations), and (ii) populations composed of one or multiple unique multilocus genotypes produced during the evolution experiment via sexual recombination of the *B. calyciflorus* species (“non‐hybrid” populations). Hybrids differed from non‐hybrids in several important traits (PGR and sexual investment). As to not obscure population responses to the experimental treatments, we analyzed hybrid and non‐hybrid populations separately.

We performed simulations to evaluate the probability that trait changes in the evolved populations resulted from selection rather than from drift. We refer to Appendix S3 for a detailed account of the simulation methodology. Briefly, we initiated neutral‐evolution simulations for a trait by assigning to 30 genotypes trait values drawn from a normal distribution with the same mean and variance as measured for the 10 seed genotypes in each of the food quality treatments. Following the design of the evolution experiment, these genotypes were used to create three identical replicate populations that were subjected to the same subsampling procedures as in the evolution and common garden experiments. All simulated genotypes were assigned the same PGR which was equal to the mean PGR of the seed genotypes during CG1 in the respective food quality treatments. We calculated trait means for the neutrally evolved populations based on genotype frequencies in the final populations. We then calculated the difference between mean traits of three simulated neutrally evolved populations and three values drawn from a normal distribution with the same mean and variance as measured for a given selection history in the common garden experiment. For each trait, this procedure was repeated 10,000 times. If 97.5% of the differences were either all larger or smaller than zero, then trait differentiation was considered greater than neutral expectations.

The effect of selection history (HP‐ vs. LP‐selected non‐hybrid populations) and its interaction with food quality (HPF vs. LPF) in the common garden experiments was tested using linear (LMM) or generalized linear mixed effect models (GLMM) depending on the error structure of the response variable. LMMs were used for PGR, elemental traits and PGR per body P:C, whereas GLMMs with binomial error and logit link were used to analyze the fraction of sexual individuals. For all response variables, population ID was used as a random factor to account for repeated measures. If the GLMM was overdispersed a replicate level random factor was included (Harrison, 2014). For PGR, we were interested in fitness response patterns concordant with local adaptation, as such we used a priori contrasts to compare HP‐ and LP‐selected non‐hybrid populations in each food treatment. Using the same model structure, a second set of analyses was performed on the same response variables to test the effect of genetic background (hybrids vs. non‐hybrids) in interaction with common garden food quality. Due to the limited number of true replicates, the response of hybrid populations from HP and LP selection regimes were combined for a comparison with non‐hybrid populations in their “home” environment.

All statistical analyses were performed in R software environment 3.6.1 (R Core Team, 2018). LMM and GLMM analyses were performed with the lme4 package (Bates et al., 2015). Statistical significances were obtained from type III sums of squares using the car package (Fox & Weisberg, 2018), LMM used Kenward‐Roger degrees of freedom. A prior and post‐hoc comparisons were performed with emmeans (Lenth et al., 2019).

## RESULTS

### Evolution experiment

HP‐selected populations showed an increase in PGR throughout the evolution experiment (*R* ^2^ = 0.20, *F*_1,76_ = 18.88, *p* < 0.001; Appendix S4: Figure S1A, Appendix S1: Table S3). In contrast, PGR in the LP‐selected populations initially declined but stabilized ~14 days into the experiment (Appendix S4: Figure S1B, Appendix S1: Table S3). In all populations, the production of resting eggs initially increased, peaking between day 5 and 10, after which it declined (Appendix S4: Figure S2). The total number of resting eggs produced during the evolution experiment did not differ significantly between the HP and LP treatments (*F*_1,12_ = 0.184, *p* = 0.676). At the experiment's conclusion, genetic diversity had been reduced to a single multilocus genotype (MLG) in 12 of the 14 populations. The two other populations were dominated by four or more MLGs (Appendix S1: Table S2). Eight of the final populations (four HP, four LP), were entirely dominated by one of two seed clones identified as hybrids. The remaining six populations (three HP, and three LP) were dominated by new sexually produced unique non‐hybrid MLGs.

### Non‐hybrid populations in common garden experiments

#### Population growth rate

**FIGURE 1:**
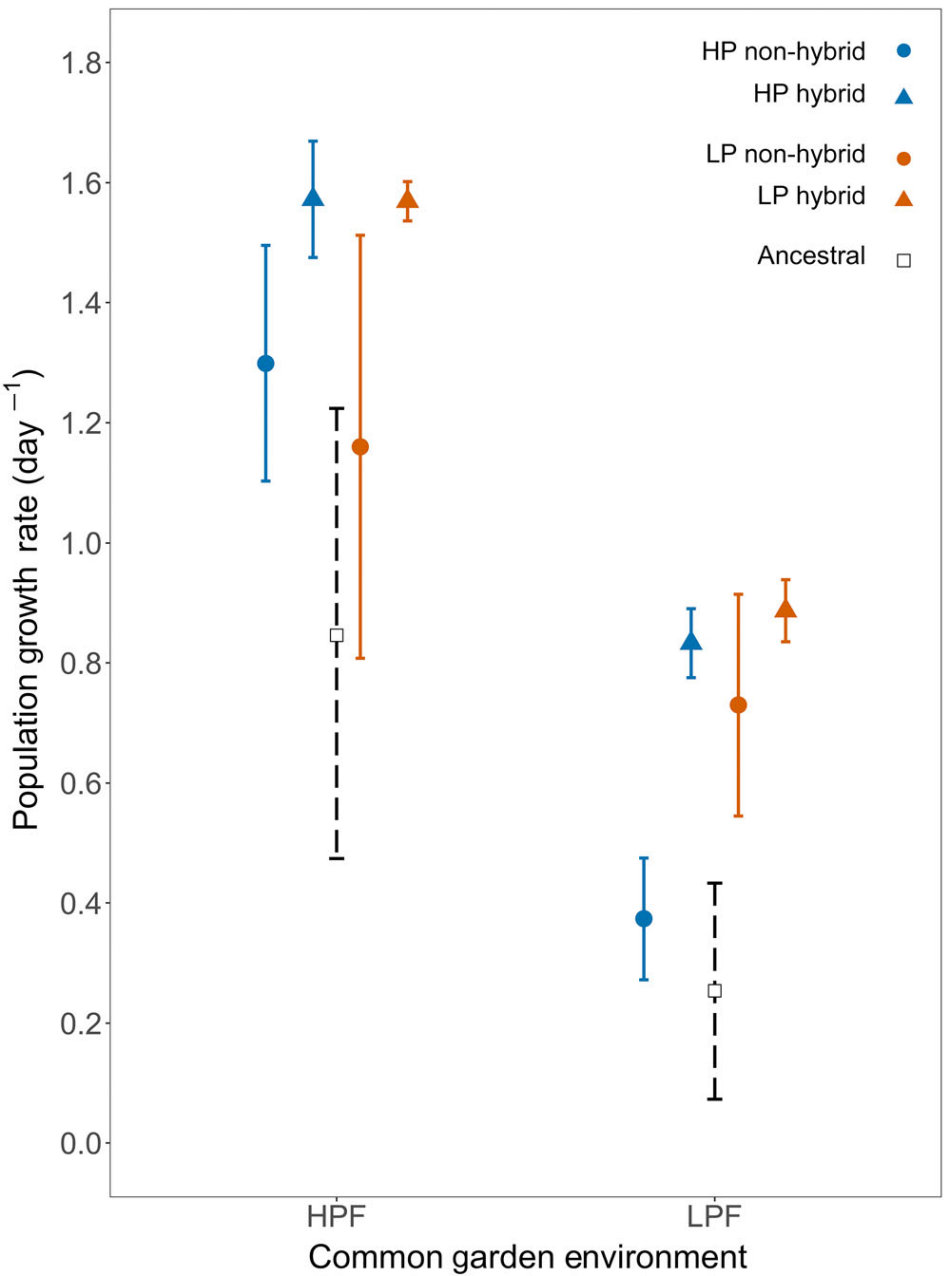
Population growth rate for evolved populations and the ancestral population in the first common garden experiment (CG1) with high (HPF) and low (LPF) phosphorus diets. During the evolution experiment, populations evolved in either a high (HP) or low (LP) phosphorus selection regime and were composed of either non‐hybrid or hybrid genotypes. For evolved populations we present means ± 2 standard errors (solid line; non‐hybrid, *n* = 3; hybrid, *n* = 2). The ancestral population means and 95% confidence intervals were obtained by bootstrapping the values observed for a subset of seed genotypes (dashed line; Appendix S1: Table S10).

In the HPF treatment, PGR of non‐hybrid populations with an HP selection history was significantly higher than the simulated neutrally evolved ancestral populations (Figure 1; Appendix S1: Table S4). Similarly, in the LPF treatment, non‐hybrid populations with an LP selection history were characterized by a significantly higher PGR than simulated ancestral populations (Figure 1; Appendix S1: Table S4).

The linear mixed model analysis revealed a substantial reduction in the PGR of all populations in the LPF compared to the HPF treatment. We also observed a significant interaction effect between selection history and common garden food treatment (Figure 1 and Table 1). In the HPF treatment, we observed no effect of selection history on PGR (a priori contrast, *p* = 0.419). Conversely, in the LPF treatment, populations with a LP selection history tended toward a greater PGR than populations with a HP selection history (a priori contrast, *p* = 0.078, Figure 1).

**TABLE 1:** Summary of linear mixed effects analyses for the population growth rate (see also Figure 1).

Genetic background and response variable	Sum Sq	Mean Sq	NumDF	DenDF	*F* value	*p*
Non‐hybrid						
Diet	5.513	5.513	1	40.00	381.78	**<0.001**
Selection History	0.007	0.007	1	4.00	0.51	0.516
Diet:SH	0.736	0.736	1	40.00	50.95	**<0.001**
Hybrid						
Diet	4.041	4.041	1	26.00	987.46	**<0.001**
Selection History	0.005	0.005	1	2.00	1.19	0.389
Diet:SH	0.007	0.007	1	26.00	1.61	0.216

*Note*: Non‐hybrid and hybrid populations were analyzed separately and the effects of diet (low or high phosphorus) and selection history (HP or LP evolved) are presented as the fixed components of the models. Bold *p*‐values are significant.

Abbreviations: DenDF, denominator degrees of freedom; Mean Sq, mean squares; NumDF, numerator degrees of freedom; *p*, *p*‐level; Sum Sq, sum of squares.

#### Population structure

In neither of the common garden food quality treatments we observed differences in the proportion of females with sexual eggs between experimental and simulated ancestral populations (Appendix S4: Figure S3, Appendix S1: Table S4).

The interaction between food quality and selection history was significant for the fraction of sexual individuals (Appendix S1: Table S5). In populations with a HP‐selection history, the fraction of sexual individuals was significantly lower in the LPF compared to the HPF common garden treatment (post hoc test, *p* = 0.006). In contrast, the faction of sexual individuals did not differ between common garden treatments in populations with a LP selection history (post hoc test, *p* = 1.00).

#### Rotifer elemental content and ratios

**FIGURE 2:**
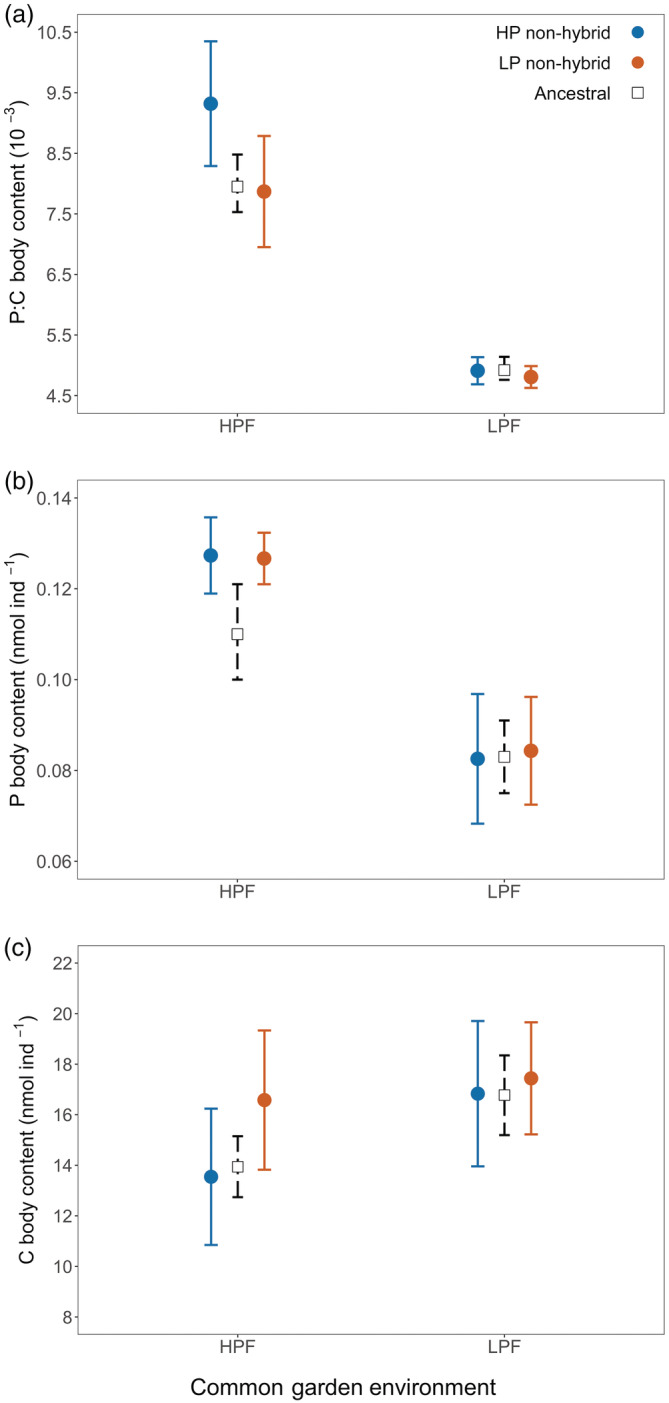
Body elemental composition for non‐hybrid evolved populations and the ancestral population in the second common garden experiment (CG2) with high (HPF) and low (LPF) phosphorus diets. During the evolution experiment, non‐hybrid populations evolved in either a high (HP) or low phosphorus (LP) selection regime. For non‐hybrid populations we present means ± 2 standard errors (solid line; *n* = 3). The ancestral population means and 95% confidence intervals were obtained by bootstrapping the values observed for a subset of seed genotypes (dashed line; Appendix S1: Table S10).

In the HPF treatment, the P:C of populations with an HP selection history was significantly higher than that of the simulated ancestral populations (Figure 2a; Appendix S1: Table S4). No such difference was found between the LP‐selected and the neutrally evolved ancestral populations in the HPF treatment (Figure 2a; Appendix S1: Table S4). In the LPF treatment, the P:C of the neutrally evolved ancestral population did not differ from populations with either selection history (Figure 2a, Appendix S1: Table S4). In the HPF treatment, populations with a HP selection history were characterized by lower body N:P than the neutrally evolved ancestral population (Appendix S4: Figure S4, Appendix S1: Table S4), while no such differences were found for the LP‐selected populations in either food treatment. We observed no differences in individual C (Figure 2c), N content (Appendix S4: Figure S4) and N:C (Appendix S4: Figure S4) between populations from the evolution experiment and neutrally evolved ancestral populations in either food treatment (Appendix S1: Table S4).

All populations in the HPF compared to the LPF treatment had significantly lower individual C content, and N:P, and higher P, N content, P:C and N:C (Figure 2 and Appendix S4: Figure S4, Appendix S1: Table S6). Populations with HP and LP selection histories did not differ in elemental content, N:P or N:C ratios in either food quality treatments. However, for individual P:C, we did observe a significant interaction between selection and common garden food treatment (Figure 2a; Appendix S1: Table S6). In the HPF treatment, the P:C of HP‐selected populations was significantly higher than that of the LP‐selected populations (post‐hoc test, *p* = 0.026); however, no such differences were observed in the LPF treatment (post‐hoc test, *p* = 0.797).

#### Population growth rate per unit body P

**FIGURE 3:**
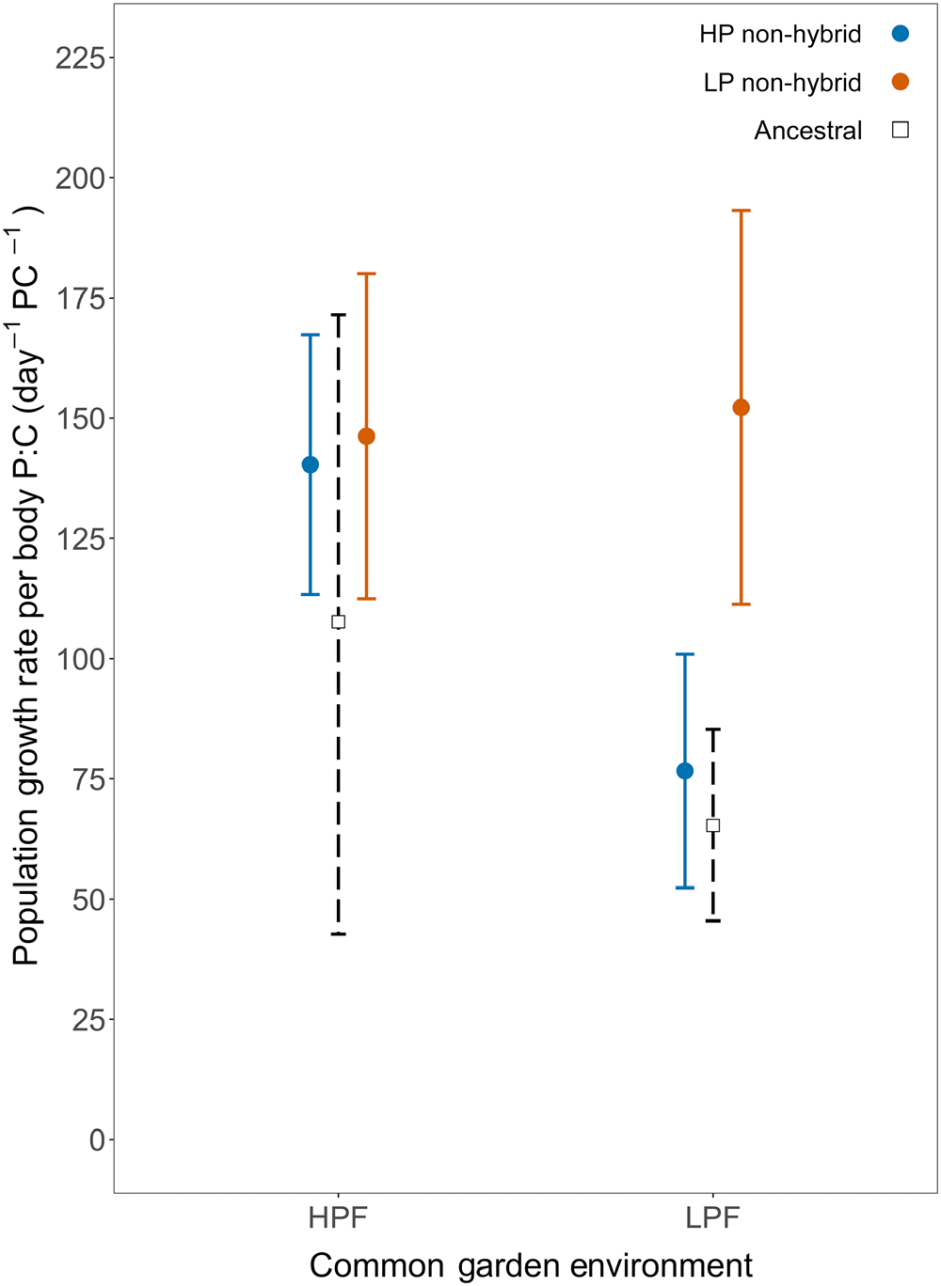
Population growth rate per body P:C for non‐hybrid evolved populations and the ancestral population with high (HPF) and low (LPF) phosphorus diets. Body P:C is expressed per individual. During the evolution experiment, non‐hybrid populations evolved in either a high (HP) or low (LP) phosphorus selection regime. For non‐hybrid populations we present means ± 2 standard errors (solid line; *n* = 3). The ancestral population means and 95% confidence intervals were obtained by bootstrapping the values observed for a subset of seed genotypes (dashed line; Appendix S1: Table S10).

In the LPF treatment, populations with an LP selection history had greater PGR per body P:C than the neutrally evolved ancestral populations (Figure 3; Appendix S1: Table S4). No such difference was found for populations with an HP selection history in the HPF treatment (Figure 3; Appendix S1: Table S4).

LP‐selected populations had significantly higher P‐use efficiency than HP‐selected populations (*p* = 0.035; Appendix S1: Table S6).

### Hybrid populations in common garden experiments

Across food quality treatments, the PGR of hybrids was significantly greater than the non‐hybrids (Figure 1; Appendix S1: Table S7) and ancestral populations (Figure 1; Appendix S1: Table S4). PGR was similar for hybrid populations regardless of selection regime in both food treatments (Figure 1, Table 1).

There was no evidence for differences in elemental content and composition between hybrids and ancestral populations, although N:P tended to be higher in hybrids (Appendix S4: Figure S4, Appendix S1: Table S4). Populations of hybrids had significantly lower P:C than non‐hybrids in both common garden treatments (Appendix S4: Figure S5, Appendix S1: Table S7). Individual C, N and P content as well as N:P and N:C did not differ between hybrid and non‐hybrid populations (Appendix S4: Figure S4 and S5, Appendix S1: Table S7).

In the first common garden experiment, hybrid populations had a strikingly lower fraction of sexual adults than non‐hybrid (Appendix S1: Table S8) and the simulated ancestral populations (Appendix S1: Table S4). In the life history experiment conducted in LPF, the propensity for sex of hybrid populations was lower than non‐hybrid (Appendix S4: Figure S5, Appendix S1: Table S9) and the simulated ancestral populations (Appendix S4: Figure S5, Appendix S1: Table S4). In CG1, on average only 5% of the mature individuals in hybrid populations were sexual compared to 30%–50% in the other populations. In the LPF life history experiment, hybrids had a 12.5% propensity for sex compared to 20%–60% in the non‐hybrid and ancestral populations (Appendix S1: Table S4).

## DISCUSSION

Our study demonstrates that the GRH has the potential to predict microevolutionary responses of populations under selection for fast growth. However, this success is largely contingent on the stoichiometric context under which such selection takes place, and the genetic background of dominant genotypes (i.e., hybrid vs. non‐hybrid). Consistent with GRH predictions, non‐hybrid populations selected for fast population growth under P‐rich conditions evolved a higher population growth rate concomitant with a higher relative body P‐content (i.e., higher P:C, lower N:P) compared to their ancestral populations. Conversely, evolution toward higher population growth rates under P‐poor conditions did not result in an increased relative P‐content. Furthermore, populations dominated by hybrid genotypes had higher PGRs than those dominated by non‐hybrids despite their lower relative P‐content across food quality treatments. Thus, although the evolutionary trajectories of non‐hybrid populations selected in HPF conditions align well with GRH predictions, it appears that increased PGRs may also be achieved through pathways that do not change body stoichiometry.

### Evolutionary response of non‐hybrid populations to a phosphorus rich diet

In non‐hybrid populations, selection for fast population growth under P‐rich conditions resulted in simultaneous evolution toward increased PGR and relative P‐content (Figures 1 and 2). These observations strongly support our GRH‐derived prediction that selection for fast population growth should promote P‐rich genotypes with high somatic growth rates. Admittedly, in this experiment, we selected for elevated population growth, and not somatic growth, due to the logistical difficulties of directly selecting on the latter (e.g., small size and rapid generation times of the model organism). However, in zooplankton in general, and *B. calyciflorus* specifically, SGR and PGR tend to covary consistently (Lampert & Trubetskova, 1996; Zhou & Declerck, 2019). Somatic P‐content is governed by P‐consumption rates (Suzuki‐Ohno et al., 2012), the efficiency of P‐assimilation (Urabe et al., 2018), and P‐retention (Frisch et al., 2014). Selection for fast population growth under HPF conditions likely selected for genotypes that were best at assimilating the abundant P of their food, and allocating it to ribosomal RNA, ultimately facilitating rapid protein synthesis.

### Evolutionary response of non‐hybrid populations to a phosphorus poor diet

When exposed to P‐deficient food resources, P‐rich, fast growing organisms have been observed to experience greater reductions in performance compared to relatively P‐poor, slow growing organisms (Sterner & Hessen, 1994). Therefore, in a P‐poor environment, genotypes that are reliant on increased P‐content to achieve elevated growth rates are not expected to be favored. Consistently, in our study, non‐hybrid populations selected in the P‐poor environment evolved increased PGR without a concomitant increase in relative P‐content in their home environment (i.e., when fed LPF).

The observed increase in PGR per unit body P:C in the P‐poor food treatment of LP‐selected populations compared to HP‐selected and ancestral populations indicates the evolution of traits other than those assumed relevant in the GRH (Figure 3). For example, under these environmental conditions, fast population growth may have been achieved through an increased metabolic use efficiency of P or by adaptations that reduce costs of excess C (Hessen & Anderson, 2008). Selection may also have benefited genotypes that are better at coping with negative indirect, non‐stoichiometric, effects of P‐limitation (Rothhaupt, 1995; Zhou et al., 2018; Zhou & Declerck, 2020). Indeed, P‐limitation of algae has been shown to strongly contribute to growth reductions in zooplankton as the result of changes in algal morphology (Van Donk et al., 1997) or biochemical quality (Muller Navarra Müller‐Navarra, 1995; von Elert et al., 2003). Although increased PGR per unit body P:C could also be generated by shifts in life history strategy, such as a reduced propensity for sex (Becks & Agrawal, 2013), our first common garden and life history experiment provided no evidence for such changes.

### Evolution of reaction norms in non‐hybrid populations

The performance of all populations was reduced under P‐poor conditions (Figure 1). Selection history, nevertheless, mediated the response of non‐hybrid populations to food quality. The reduction in population growth rate in the P‐poor compared to the P‐rich environment was much smaller for the populations with an LP selection history (37%) compared to populations with an HP selection history (69%). Contrary to the idea of local adaptation, in the P‐rich common garden environment populations with a LP selection history did not underperform compared to populations with an HP selection history, suggesting the absence of a trade‐off.

Overall, performance differences among populations in their home environments (i.e., LP and HP adapted populations in LPF and HPF treatments, respectively) were less pronounced than expected based on the negative effect of P‐limitation. This was entirely due to the evolutionary response of the LP‐adapted populations. Instead of local adaptation, we observed a pattern indicative of counter gradient variation with a strong genotype by environment interaction (Conover et al., 2009). Zooplankton populations have shown similar evolutionary responses to P‐limitation. For example, Frisch et al. (2014) resurrected genotypes from periods of high and low resource availability. Genotypes originating from oligotrophic periods before European settlement had higher growth performances under P‐poor conditions than genotypes from recent, more eutrophic periods. In contrast, no consistent differences between genotypes were found in P‐rich food. Declerck et al. (2015) performed an evolution experiment with rotifers that selected for competitive ability under LPF and HPF conditions. They demonstrated that when exposed to a LPF treatment, populations with an LP selection history realized a higher food exploitation efficiency than populations with an HP selection history. Conversely, in the HPF treatment, LP‐adapted populations showed a similar performance as HP‐adapted populations. Overall, the remarkable consistency of reaction norm responses of zooplankton consumers to P‐limitation, despite widely different contexts, suggests that adaptation to P‐limitation may represent a relatively general but underappreciated example of cryptic evolution in zooplankton (Kinnison et al., 2015). Furthermore, it is remarkable that none of these studies on adaptation to P‐limitation have found trade‐offs with performance under P‐sufficient conditions.

### Hybrid populations

Hybrid populations had greater PGRs than ancestral and most non‐hybrid populations under all conditions. Higher PGRs were more strongly associated with a lower propensity for sex than with an increase in relative P‐content (Appendix S4: Figure S5A,E). In rotifers, a reduction in the propensity for sex enhances clonal PGRs by reducing demographic costs associated with sexual reproduction (Becks & Agrawal, 2013; Serra & Snell, 2009). The fact that hybrids did not dominate all populations despite their relatively high PGRs is likely because stochasticity had an important role in determining the genotypic composition of the populations of our evolution experiment. Nevertheless, our hybrid results demonstrate that PGRs may be determined by traits other than individual relative P‐content, and illustrate some limitations of the GRH as a predictive microevolutionary framework.

### Ecological implications of rapid adaptation to selection for fast growth and its dependency on stoichiometric context

Herbivores are important for trophic dynamics (Trussell & Schmitz, 2012). Experiments have revealed that microevolutionary adaptation in consumer populations can affect higher trophic levels (e.g., Fryxell et al., 2019); however, the eco‐evolutionary implications of adaptation in a stoichiometric context has received limited consideration (Yamamichi et al., 2015). Humans are increasingly impacting nutrient supply rates and the stoichiometry of primary producers in aquatic systems (Smith & Schindler, 2009; Stockner et al., 2000). To improve our understanding of anthropogenic impacts on food web ecology, further integration of stoichiometry and the study of eco‐evolutionary dynamics is needed, including feedbacks between trophic levels (Hall, 2009; Wood et al., 2018).

The results of this study demonstrate the predictive power of the GRH. We observed that, in laboratory conditions that select for fast population growth, where P is not limiting, and population growth is density independent (i.e., resources are non‐limiting), increases in population growth rate are concomitant with increases in relative P‐content. Such conditions are similar to what natural consumer populations may be experiencing when colonizing novel habitats (MacArthur & Wilson, 1967), or when being exposed to high levels of predation (Walsh & Reznick, 2008), short growing seasons (Elser et al., 2000; Walsh & Post, 2011), or frequent disturbances (e.g., droughts, disease outbreaks; Lachish et al., 2009; Vanschoenwinkel et al., 2010). Our observation of rapid evolutionary responses by consumers to such a selection regime may have important yet understudied eco‐evolutionary implications for higher trophic levels and food web functioning (e.g., by enhancing secondary productivity). For example, increased PGRs in primary consumers may at first partially compensate for mortality rates imposed by predators. Simultaneously, increases of herbivore relative P‐content, concomitant with their increased productivity, may contribute to an increased resource base for predators. The resulting enhancement of predator productivity (Boersma et al., 2008; Malzahn et al., 2010; Schoo et al., 2012) may ultimately result in a strengthened top‐down control of primary consumers (Hall et al., 2007). To explore the eco‐evolutionary consequences of the adaptive responses observed in our study and its dependency on stoichiometric context, there is clearly a need for more dedicated experiments and modeling efforts.

## CONCLUSION

Our results demonstrate the validity of the GRH as a framework for the prediction of microevolutionary responses of populations to selection for fast growth. At the same time, they also demonstrate the limits of its application, as predictions proved strongly dependent on the environmental context and genetic background of genotypes under consideration. Although the evolution of higher PGRs coincided with increased individual relative P‐content for populations selected with HPF resources, the evolution of higher PGRs in hybrid populations and those selected with LPF resources was achieved through other mechanisms. This study provided resources ad libitum, as such negative feedbacks between the consumer population growth and its resources were negligible. Further investigations are needed to evaluate the application of the GRH as a predictive framework in other environmental contexts such as when there is competition for resources.

This study clearly demonstrates the importance of stoichiometric context to evolutionary processes (Kay et al., 2005). In experimental work, the selection history of genotypes is almost never considered although it may be pivotal in explaining apparent discrepancies between different studies (e.g., DeMott et al., 1998; Hood & Sterner, 2014; Sherman et al., 2017). For this reason, we advocate for the inclusion of organismal selection histories into the ecological stoichiometric framework whenever possible. Furthermore, the observation of rapid evolution in both body stoichiometry and population demography in this study suggests the need for more investigations of the impact of both apparent and cryptic evolution in herbivore consumers on trophic dynamics.

## CONFLICT OF INTEREST

The authors declare no conflict of interest.

## Supporting information


Appendix S1

Appendix S2

Appendix S3

Appendix S4

## Data Availability

Data (Lemmen et al., 2022a) are available in Dryad at https://doi.org/10.5061/dryad.8gtht76r8. Code for the simulation of neutral evolution in the ancestral populations and the statistical analysis (Lemmen et al., 2022b) is available in Zenodo at https://doi.org/10.5281/zenodo.6341155.
