# Supplementary material for: An experimental test of the growth rate hypothesis as a predictive framework for microevolutionary adaptation
Source: Ecology. 2022 Oct 23;104(1):e3853. doi: 10.1002/ecy.3853 (PMC10078216; doi:10.1002/ecy.3853)
Supplement: Supplementary file 1 — Appendix S1 [file ECY-104-0-s004.pdf]

Supporting information

Title: An experimental test of the growth rate hypothesis as a predictive framework for microevolutionary adaptation

Authors: Kimberley D. Lemmen, Libin Zhou, Spiros Papakostas, and Steven A.J. Declerck

Journal: Ecology

Appendix S1 Supplementary Tables

Table S1. Identifying characteristics of the seed genotypes used to initiate the evolution experiment. The 30 genotypes originated from seven locations throughout The Netherlands and are identified by a unique Clone ID. Species identity and the multilocus genotype (MLG) was determine by microstatellite analysis (Appendix S1). Clones identified as hybrids are hybrids between the cryptic species *B. calyciflorus* and *B. elevatus* (Michaloudi *et al.* 2018) following the observations of Papakostas *et al.* (2016). The MLG for the clonal lines are described by the size in base pairs (bp) of alleles (separated with "/") at 11 microsatellite loci using the primers SSR1 and SSR2 as described in *Declerck et al.* (2015).

					Multilocus Genotype										
					SSR1						SSR2				
Pond ID	Latitude	Longitude	Clone ID	Species	A1	A5	A9	A11	A14	A15	A3	A4	A12	A8	A7
7	51.854065°	5.893175°	7-I	Hybrid	134/143	98/102	161/175	111/113	99/99	78/87	121/121	99/105	147/149	235/235	183/183
			7-II	*B. calyciflorus*	143/143	96/98	163/163	109/109	99/99	87/90	121/121	105/105	149/149	229/235	179/183
			7-III	*B. calyciflorus*	146/146	96/98	163/163	109/111	99/99	87/90	121/121	105/105	149/149	229/235	179/183
			7-IV	Hybrid	134/143	98/102	163/175	109/113	99/99	78/87	121/121	93/99	147/147	232/232	179/179
69	52.090694°	4.338444°	69-I	*B. calyciflorus*	134/146	98/98	163/163	109/109	93/99	93/102	125/125	105/105	149/149	238/238	179/183
			69-II	*B. calyciflorus*	143/143	98/98	163/163	109/111	99/99	81/102	123/123	93/105	149/149	229/229	179/183
			69-III	Hybrid	134/143	100/102	163/175	109/113	99/99	78/90	121/125	99/105	147/147	238/238	179/179
			69-IV	Hybrid	134/143	100/102	163/175	109/113	99/99	78/90	121/125	99/105	147/147	238/238	179/179
102	52.0263°	4.18355°	102-12	*B. calyciflorus*	143/143	98/98	163/165	109/111	93/99	87/90	123/123	105/105	147/149	na	179/185
			102-17	*B. calyciflorus*	143/143	98/98	163/165	109/111	93/99	87/90	121/125	105/105	147/149	229/229	181/181
			102-102	*B. calyciflorus*	143/143	98/98	163/163	109/109	99/99	81/90	121/123	93/105	147/149	235/235	179/179
			102-120	*B. calyciflorus*	143/143	96/98	163/163	109/109	99/99	87/90	119/119	na	147/149	229/229	181/185
			102-127	Hybrid	134/134	98/102	165/175	109/113	99/99	78/90	121/125	96/99	147/147	238/238	185/185
118	52.935474°	5.692689°	118-25	*B. calyciflorus*	143/143	96/98	163/165	109/109	93/99	90/90	125/125	99/105	147/147	235/238	185/185
			118-26	*B. calyciflorus*	143/149	96/96	163/163	109/109	90/93	93/93	125/125	105/105	147/149	238/238	179/185
			118-29	*B. calyciflorus*	143/143	98/98	168/165	109/111	93/99	87/90	121/125	105/105	147/149	na	179/179
			118-30	*B. calyciflorus*	143/143	98/98	163/163	109/109	96/99	90/93	125/125	93/102	149/149	229/229	185/185
			118-34	*B. calyciflorus*	134/143	96/96	163/163	111/111	90/99	81/90	125/125	93/105	147/149	229/229	179/185
128	52.640324°	4.730287°	128-04	*B. calyciflorus*	143/143	98/98	163/163	111/111	99/99	93/93	121/125	na	147/147	238/238	183/183
			128-I	*B. calyciflorus*	134/143	98/98	163/163	109/111	96/99	81/81	125/125	93/105	147/149	238/238	179/179
			128-II	*B. calyciflorus*	143/143	96/98	163/163	109/109	99/99	81/90	125/125	105/105	147/147	238/238	179/179
			128-III	Hybrid	134/146	96/102	163/175	111/113	93/93	78/90	121/121	99/105	147/149	232/232	183/183
			128-IV	*B. calyciflorus*	140/140	96/96	163/163	109/109	99/99	93/93	125/125	105/105	147/147	229/229	183/183
168	51.491446°	4.3068°	168-03	*B. calyciflorus*	143/143	98/98	163/163	109/109	96/96	87/90	125/125	105/105	147/147	238/238	179/179
			168-05	*B. calyciflorus*	143/143	96/98	163/163	109/109	93/93	87/87	125/125	93/102	147/147	235/235	179/185
			168-08	*B. calyciflorus*	143/143	96/98	163/163	109/109	93/93	87/87	125/125	93/102	147/147	235/235	179/185
			168-16	Hybrid	143/143	98/102	163/163	109/111	93/93	78/90	123/123	93/99	147/147	229/229	179/185
			168-19	*B. calyciflorus*	143/143	98/98	163/163	109/109	96/96	90/93	125/125	99/102	147/147	238/238	179/179
180	51.815501°	4.031201°	180-01	*B. calyciflorus*	143/143	100/100	163/163	109/109	93/93	81/90	125/125	93/105	147/149	238/238	179/185
			180-02	*B. calyciflorus*	143/143	96/98	163/163	109/109	84/93	87/90	125/125	96/105	147/149	na	183/185

Table S2. Multilocus genotype (MLG) composition of the populations at the conclusion of the evolution experiment as determined by microsatellite analysis. Each replicate population is represented by a unique ID and its use in either the first (CG1) or second (CG2) common garden experiment is denoted with an "X". Clones identified as hybrids between the cryptic species *B. calyciflorus* and *B. elevatus* (Michaloudi *et al.* 2018) were already present as seed clones in the ancestral populations. Each MLG is described by the size in base pairs (bp) and each allele is separated with "/", using primers SSR1 and SSR2 as described in Declerck *et al.* (2015). PROPN indicated the proportion of the population represented by a given genotype.

							Multilocus Genotype										
							SSR1						SSR2				
Selection History	ID	CG1	CG2	Species	Seed Genotype	PROPN	A1	A5	A9	A11	A14	A15	A3	A4	A12	A8	A7
High Phosphorus	**HP1**	X	X	*B. cal*		28%	143/143	96/96	163/163	109/109	96/99	87/93	123/125	102/105	147/149	235/235	181/185
				B. cal		11%	143/143	96/96	163/163	109/109	96/99	87/93	125/125	102/105	147/149	235/235	179/185
				B. cal		11%	143/143	96/96	163/163	109/109	96/99	87/93	125/125	93/105	147/149	235/238	179/185
				B. cal		22%	143/143	96/96	163/163	109/109	96/99	87/93	123/125	93/105	149/149	235/238	185/185
				B. cal		28%	143/143	96/96	163/163	109/109	96/99	87/93	125/125	93/105	149/149	235/238	185/185
	**HP2**	X	X	*B. cal*		100%	143/143	96/96	163/163	109/109	96/99	87/90	123/125	93/105	147/147	235/238	179/179
	HP3			Hybrid	128-III	100%	134/146	96/102	163/175	111/113	96/96	78/90	121/121	99/105	147/149	232/232	183/183
	**HP4**	X	X	*B. cal*		100%	143/149	96/98	163/163	109/109	96/99	87/90	125/125	93/105	149/149	235/235	185/185
	**HP5**	X	X	Hybrid	128-III	100%	134/146	96/102	163/175	111/113	96/96	78/90	121/121	99/105	147/149	232/232	183/183
	HP6			Hybrid	128-III	100%	134/146	96/102	163/175	111/113	96/96	78/90	121/121	99/105	147/149	232/232	183/183
	HP7	X		Hybrid	128-III	100%	134/146	96/102	163/175	111/113	96/96	78/90	121/121	99/105	147/149	232/232	183/183
Low Phosphorus	**LP1**	X	X	*B. cal*		100%	143/143	96/98	163/163	109/109	99/99	90/90	121/125	93/105	149/149	235/238	181/185
	**LP2**	X	X	*B. cal*		100%	143/149	96/98	163/163	109/109	99/99	90/90	121/123	105/105	147/149	na	179/179
	**LP3**	X	X	Hybrid	69-III/IV	100%	134/143	100/102	163/175	109/113	99/99	78/90	121/125	99/105	147/147	238/238	179/179
	LP4			Hybrid	128-III	100%	134/146	96/102	163/175	111/113	93/93	78/90	121/121	99/105	147/149	232/232	183/183
	LP5			Hybrid	69-III/IV	100%	134/143	100/102	163/175	109/113	99/99	78/90	121/125	99/105	147/147	238/238	179/179
	LP6	X		Hybrid	69-III/IV	100%	134/143	100/102	163/175	109/113	99/99	78/90	121/125	99/105	147/147	238/238	179/179
	**LP7**	X	X	Hybrid	128-III	40%	134/146	96/102	163/175	111/113	96/96	78/90	121/121	99/105	147/149	232/232	183/183
				B. cal		20%	140/146	96/98	163/163	109/111	99/99	90/93	121/125	105/105	147/149	na	179/183
				B. cal		25%	134/140	96/98	163/163	109/113	99/99	90/93	121/125	105/105	149/149	232/232	183/183
				B. cal		15%	140/140	98/98	163/163	109/109	99/99	93/93	121/121	105/105	149/149	235/235	179/183

Table S3. Comparison of linear and piecewise (PW) regression models describing the course of population growth rate over time in the evolution experiment for populations in low and high phosphorus food treatments (n=7 populations). Models in bold are those selected for interpretation based on AIC. Davies test p-value indicates a significant difference between the two slopes of the piecewise regression models.

						Segment One		Segment Two		
**Food Quality**	**Model**	**df**	**AIC**	**Segmented at**	**Confidence interval**	**Slope**	**95% CI**	**Slope**	**95% CI**	**Davies test p-value**
High Phosphorus	**Linear**	**3**	**-7.15**	**NA**	**NA**	**0.01**	**(0.005,0.014)**	**NA**	**NA**	**NA**
	PW	5	-6.08	5	(0,12)	-0.04	(-0.157,0.080)	0.012	(0.006,0.018)	0.293
Low Phosphorus	Linear	3	-56.79	NA	NA	-0.003	(-0.007,0)	NA	NA	NA
	**PW**	**5**	**-69.79**	**14**	**(9,18)**	**-0.025**	**(-0.039,-0.011)**	**0.006**	**(0,0.012)**	**<0.001**

Table S4. Means of trait values simulated for neutrally evolved ancestral populations, with 2.5 and 97.5 percentiles. For the HP-selected (n=3), LP-selected (n=3), and hybrid (n=4) populations we also present the mean value of the traits observed in the common garden experiments and the significance (p-value) of differences between observed trait means and simulated means for neutrally evolved ancestral populations, as explained in Appendix S2. Traits were measured in the following units: Population growth rate (day^{-1}), fraction of sexual individuals (% of mature population), C, N and P content (nmol $individual^{-1}$), elemental ratios are unitless, population growth rate per body P (day^{-1} nmol P^{-1}), and propensity for sex (% of total population).

		Neutrally Evolved			HP-selected		LP-selected		Hybrid	
Trait		Mean	2.5 Percentile	97.5 Percentile	Mean	p-value	Mean	p-value	Mean	p-value
Population Growth Rate										
	HPF	0.848	0.3468	1.218	1.299	**0.037**	1.160	0.222	1.571	**0.001**
	LPF	0.263	0.078	0.450	0.374	0.297	0.730	**< 0.001**	0.860	**< 0.001**
Fraction Sexual Individuals										
	HPF	0.515	0.379	0.647	0.401	0.207	0.316	0.372	0.048	**< 0.001**
	LPF	0.250	0.118	0.384	0.194	0.575	0.314	0.609	0.021	**0.001**
C										
	HPF	13.894	12.712	15.096	13.544	0.8112	16.579	0.073	15.428	0.575
	LPF	16.816	15.349	18.274	16.833	0.994	17.439	0.621	16.802	0.994
N										
	HPF	2.733	2.515	2.955	2.670	0.806	3.226	0.053	3.009	0.628
	LPF	2.461	2.278	2.642	2.454	0.9778	2.583	0.475	2.364	0.783
P										
	HPF	0.111	0.100	0.121	0.127	**0.014**	0.127	**0.007**	0.123	0.519
	LPF	0.083	0.076	0.090	0.083	0.978	0.084	0.805	0.083	0.985
PC										
	HPF	0.0080	0.0075	0.0085	0.0093	**0.017**	0.0078	0.874	0.0080	0.849
	LPF	0.0049	0.0047	0.0051	0.0049	0.952	0.0048	0.367	0.0045	0.080
NC										
	HPF	0.197	0.193	0.200	0.198	0.737	0.194	0.584	0.194	0.422
	LPF	0.147	0.142	0.151	0.146	0.883	0.148	0.524	0.140	0.140
NP										
	HPF	24.957	23.474	26.463	21.517	**0.001**	24.928	0.960	24.267	0.523
	LPF	29.556	28.712	30.396	29.922	0.775	31.010	0.187	30.883	**0.045**
PGR per PC										
	HPF	107.671	42.731	171.483	140.346	0.346	146.237	0.288	200.200	**0.006**
	LPF	65.338	45.466	85.300	76.645	0.471	152.227	**< 0.001**	188.712	**< 0.001**
Propensity for Sex										
	LPF	0.509	0.416	0.603	0.574	0.426	0.278	0.195	0.125	**< 0.001**

Table S5. Summary of generalized linear mixed model analysis testing for non-hybrid populations on the effects of diet and selection history on the fraction of sexual idividuals in the first common garden experiment (CG1). Effects of common garden diet (LPF or HPF) and population selection history (LP or HP evolved) are presented as the fixed components of the models. The fraction of sexual females was calculated as the number of females with sexual eggs (male and diapausing eggs) divided by the total number of mature individuals (i.e., adults with male, diapausing or amictic eggs). Significant effects are in bold (see also Figure S3).

	**Chisq**	**DF**	**p**
Fraction of Sexual Individuals			
Diet	3.45	1	**0.063**
Selection History	0.002	1	0.989
Diet:SH	9.301	1	**0.002**

Table S6. Effects of diet and selection history on the elemental content and elemental ratios of non-hybrid populatons from the evolution experiment. Data were obtained from the second common garden experiment and analysed with linear mixed models where diet and selection history were treated as fixed factors. Significant effects are in bold. See Table 1 for abbreviations.

	Sum SqŦ	Mean SqŦ	NumDFŦ	DenDFŦ	F value	pŦ
P:C						
Diet	1.13E-04	1.13E-04	1	25.3	220.26	**< 0.001**
Selection History	2.30E-06	2.30E-06	1	4.0	4.47	0.103
Diet:SH	2.66E-06	2.66E-06	1	25.3	5.15	**0.032**
N:P						
Diet	4.30E+02	4.30E+02	1	25.4	81.71	**< 0.001**
Selection History	2.46E+01	2.46E+01	1	4.1	4.67	0.095
Diet:SH	7.22E+00	7.22E+00	1	25.4	1.37	0.252
N:C						
Diet	1.97E-02	1.97E-02	1	29.0	262.81	**< 0.001**
Selection History	7.27E-07	7.27E-07	1	29.0	0.01	0.922
Diet:SH	2.72E-05	2.72E-05	1	29.0	0.36	0.552
P content						
Diet	1.71E-02	1.71E-02	1	28.0	63.69	**< 0.001**
Selection History	1.91E-06	2.80E-06	1	4.0	0.01	0.937
Diet:SH	1.34E-05	1.34E-05	1	28.0	0.05	0.825
C content						
Diet	3.75E+01	3.75E+01	1	25.0	6.98	**0.014**
Selection History	4.99E+00	4.99E+00	1	3.9	0.93	0.391
Diet:SH	9.39E+00	9.39E+00	1	25.0	1.75	0.198
N content						
Diet	1.45E+00	1.45E+00	1	25.1	9.02	**0.006**
Selection History	2.25E-01	2.25E-01	1	3.9	1.40	0.303
Diet:SH	2.78E-01	2.78E-01	1	25.1	1.73	0.201
PGR per P:C						
Diet	6.09E+03	6.09E+03	1	8.0	3.22	0.111
Selection History	5.20E+01	5.20E+01	1	8.0	6.41	**0.035**
Diet:SH	3.64E+03	3.64E+03	1	8.0	4.69	0.062

Table S7. Summary of linear mixed effects analyses comparing traits of non-hybrid and hybrid populations based on data from the first and second common garden experiments. Effects of diet (LPF or HPF) and genetic background (non-hybrid or hybrid) are presented as the fixed components of the models. Significant effects are in bold (see also Figure S5). See Table 1 for abbreviations.

	**Sum SqŦ**	**Mean SqŦ**	**NumDFŦ**	**DenDFŦ**	**F value**	**pŦ**
Population Growth Rate						
Diet	9.39E-01	9.39E-01	1	8.1	147.29	**< 0.001**
Genetic Background	4.11E-02	4.11E-02	1	6.6	6.45	**0.041**
Diet:Genetic Background	1.15E-02	1.15E-02	1	8.1	1.80	0.216
P:C						
Diet	1.04E-04	1.04E-04	1	24.0	169.17	**< 0.001**
Genetic Background	3.71E-06	3.71E-06	1	24.0	6.07	**0.021**
Diet:Genetic Background	1.29E-06	1.29E-06	1	24.0	2.10	0.160
N:P						
Diet	4.27E+02	4.27E+02	1	24	76.712	**< 0.001**
Genetic Background	1.08E+01	1.08E+01	1	24	2.0824	0.161
Diet:Genetic Background	1.10E+01	1.10E+01	1	24	2.0664	0.164
N:C						
Diet	1.78E-02	1.78E-02	1	24	202.19	**< 0.001**
Genetic Background	2.56E-04	2.56E-04	1	24	2.9139	0.101
Diet:Genetic Background	5.75E-05	5.75E-05	1	24	0.6542	0.427
P content						
Diet	6.65E-03	6.65E-03	1	12.5	20.86	**0.001**
Genetic Background	1.07E-05	1.07E-05	1	5.1	0.03	0.862
Diet:Genetic Background	7.70E-06	7.70E-06	1	12.5	0.02	0.879
C content						
Diet	2.03E+01	2.03E+01	1	10.2	3.79	0.080
Genetic Background	3.93E-01	3.93E-01	1	4.7	0.07	0.798
Diet:Genetic Background	4.34E+00	4.34E+00	1	10.2	0.81	0.388
N content						
Diet	3.97E-01	3.97E-01	1	10.2	2.37	0.155
Genetic Background	3.92E-03	3.92E-03	1	4.8	0.02	0.885
Diet:Genetic Background	2.04E-01	2.04E-01	1	10.2	1.22	0.296

Table S8. Summary of generalized linear mixed model analyses comparing non-hybrid and hybrid populations in the first common garden. The fraction of sexual females was calculated as the number of females with sexual eggs (male and diapausing eggs) divided by the total number of mature individuals (i.e., adults with male, diapausing or amictic eggs). Effects of diet (low or high phosphorus) and genetic background (non-hybrid or hybrid) are presented as the fixed components of the models. Significant effects are in bold (see also Figure S5E).

	Chisq	DF	p
Fraction of Sexual Individuals			
Diet	4.789	1	**0.029**
Genetic Background	68.023	1	**< 0.001**

Table S9. Summary of generalized linear model analyses of the effects of genetic background (non-hybrid or hybrid) in the LPF life history experiment. The propensity for sex was calculated as the number of sexual indiviuals divided by the total number of mature individuals (i.e., sexual, asexual or dead). Significant effects are in bold.

	Chisq	Z-value	p
Propensity for Sex			
Genetic Background	11.576	1	**< 0.001**

Table S10. Seed genotypes used to calculate the ancestral population means and 95% confidence intervals (for methodology see Appendix S2). Clone ID matches values in Table S1. 'Dryad ID' refers to the numerical ID associated with each clone in the online dataset. An 'X' indicates the clone was part of the corresponding experiment and the observed response was used to calculate the ancestral population means and 95% confidence intervals.

Clone ID	Dryad ID	Common Garden 1	Common Garden 2	Life History
168-3	1			
168-5	2	X	X	X
168-8	3			
168-16	4			
168-19	5	X	X	X
180-1	6			
180-2	7	X		
118-20	8			
118-25	9	X		
118-26	10	X		
118-30	11		X	X
118-34	12			
102-12	13	X	X	X
102-17	14			
102-102	15		X	X
102-120	16			
102-127	17			
128-4	18			
128-I	19			
128-II	20	X	X	X
128-III	21		X	X
128-IV	22			
69-I	23			
69-II	24	X		
69-III	25			
69-IV	26			
7-I	27			
7-II	28	X	X	X
7-III	29			
7-IV	30	X	X	X

Literature Cited

Michaloudi, E., Papakostas, S., Stamou, G., Neděla, V., Tihlaříková, E., Zhang, W., *et al.* (2018). Reverse taxonomy applied to the *Brachionus calyciflorus* cryptic species complex: Morphometric analysis confirms species delimitations revealed by molecular phylogenetic analysis and allows the (re)description of four species. *PLoS One*, 13, e0203168.

Papakostas, S., Michaloudi, E., Proios, K., Brehm, M., Verhage, L., Rota, J., *et al.* (2016). Integrative taxonomy recognizes evolutionary units despite widespread mitonuclear discordance: Evidence from a rotifer cryptic species complex. *Syst. Biol.*, 65, 508–52
