# Supplementary material for: An experimental test of the growth rate hypothesis as a predictive framework for microevolutionary adaptation
Source: Ecology. 2022 Oct 23;104(1):e3853. doi: 10.1002/ecy.3853 (PMC10078216; doi:10.1002/ecy.3853)
Supplement: Supplementary file 2 — Appendix S2 [file ECY-104-0-s003.pdf]

Supporting information

Title: An experimental test of the growth rate hypothesis as a predictive framework for microevolutionary adaptation

Authors: Kimberley D. Lemmen, Libin Zhou, Spiros Papakostas, and Steven A.J. Declerck

Journal: Ecology

Appendix S2 Additional Methodological Information

Maintenance of Seed Genotype Batch Cultures

We maintained seed genotypes in 1.5 mL wells of tissue culture plates under constant light and at room temperature throughout the duration of the study. Theses cultures had a density of 10-15 rotifer individuals/mL, and we provided them 1 mL of an algal suspension comprised of nutrient replete *Chlamydomonas reinhardtii* and nutrient-free COMBO media (Kilham *et al.* 1998) at a concentration of ~1000 μmol L^{-1} C. Three times a week, we moved ten individuals from each culture into new algae suspension.

Microstatellite Analysis

Rotifer genotyping was performed on DNA extracts from single rotifers using the HotSHOT method (Montero-Pau *et al.* 2008). In each panel we used, for a 5 μl PCR reaction, 0.5 μl template DNA, 0.1 μl from each primer (from 10 pmol/μl concentration), (2.5 μl Master Mix of the QIAGEN Multiplex PCR kit (2× stock concentration), and 0.8 μl Milli-Q water. PCR thermocycler conditions involved an initial denaturation of 95°C for 15 min required to activate the HotStarTaq DNA polymerase of the QIAGEN Multiplex PCR kit. The next step was 30 cycles of 30 sec at 95°C, 90 sec at 56°C, and 60 sec at 72°C. A final elongation step of 30 min at 60°C completed the amplification. The PCR amplicon was then diluted 1:20 with Milli-Q water and 1 μl from this dilution was then mixed with 8.8 μl of formamide and

0.2 µl of GeneScanTM 500 LIZTM size standard (Applied Biosystems, CA) prior to loading 1 µl into an ABI Prism 3130 DNA Analyzer (Applied Biosystems, CA). Samples were run for 30 min at 15000V using 36 cm capillaries. Allele calling was performed with the software GeneMapper® v. 4.0 using GS500 (-250) LIZ as an option for size standard. Peak threshold was set to 50 rfu for each dye but two different users also did manual inspection and correction, when necessary, of the allele calls. Multilocus genotype (MLG) assignment and analyses of genetic relationship were conducted with GenoDive v.2.b27 (Meirmans & Van Tienderen 2004), a program designed for the analysis of genetic diversity of clonal organisms.

Before the evolution experiment, we performed a microsatellite analysis on two individuals from each of the thirty seed genotypes to establish their multilocus genotype (MLG) using the microsatellite primers as described in Declerck *et al*. (2015). At the end of the evolution experiment (day 35), we extracted DNA from ten haphazardly chosen individuals from each of the experimental populations. We then performed microsatellite analysis to determine the genetic composition of each population. If we detected more than one MLG within a population (i.e., HP1 and LP7) an additional ten individuals were sequenced.

Algae Cultures and Food Preparation

We used the green algae *Chlamydomonas reinhardtii* as food source for the rotifers. To produce phosphorus rich algae ('HPF': molar C:P ratio 121 ± 11.9SE) we used media with 65 µmol L^{-1} P under ≈40 µmol quanta m^{-2} s^{-1} of continuous light, while phosphorus poor algae ('LPF': molar C:P 671 ± 9.9SE) received media with 15 µmol L^{-1} P and under ≈120 µmol quanta m^{-2} s^{-1} of continuous light. For batch cultures and all experiments, we prepared the algal suspension by estimating carbon content using biovolume (Multisizer 3 Coulter Counter, Beckman Coulter) and diluting the algae with nutrient-free COMBO media to the

desired concentration. After dilution, we added a vitamin mixture (Kilham *et al.* 1998) at a concentration of 1 mL L^{-1}.

Demographic Classification

To assess population demography we counted preserved samples for each replicate of CG1 using a MZ16 Leica stereomicroscope at 25X magnification. Individuals were classified as one of the following: females without eggs, females with sexual eggs (male eggs and diapausing eggs), and females carrying parthenogenetic eggs. We also recorded the total number of parthenogenetic and diapausing eggs (loose and attached to female).

Rotifer Culturing for Quantification of Elemental Composition (CG2)

We used a different culturing method in CG2, as quantifying rotifer elemental body composition requires many individuals in the same body condition. In both common gardens we allowed populations to grow exponentially under *ad libitum* food concentrations. In CG1 this was achieved in a constant culture volume by daily reducing population densities so that food did not become limiting. In CG2, this was achieved by allowing population size to grow while increasing the culture volume proportionally on a daily basis so that population density remained constant (20 rotifers/mL) and food abundant.

To initiate CG2 we used individuals from the cultures that were maintained throughout the evolution experiment and CG1. Sixty individuals from each evolved population and seed genotype were randomly allocated to either an HP- or LP-food culture. Initially, we provided all experimental units with 3mL of food suspension at a concentration of 1550 μmol L^{-1} C. We assessed population size daily by taking a 1mL sample and counting all individuals present. Population size was then used to determine the subsequent culture volume and individuals were transferred to the fresh medium using an 80μm mesh. In this way, we scaled

up culture volumes until we reached a population size of 4000 individuals. We sampled populations for elemental quantification after three consecutive days of constant population growth while keeping population densities constant. We obtained replicates by subsampling 100 individuals from the large population and restarting the culture. We waited at least seven days between sampling replicates, allowing for three asexual generations at a minimum, thereby reducing the impact of maternal effects and allowing for replicates to become independent. All populations and seed clones were represented by three replicates.

Determination of Elemental Composition

We determined rotifer C and N contents using a FLASH 2000 organic element analyzer (Interscience B.V., Breda, Netherlands), and P-content with a QuAAtro segmented flow autoanalyzer (Beun de Ronde, Abcoude, Netherlands). For each of these analyses we used a sample of 100 individuals with a single parthenogenetic egg. Prior to harvesting for elemental analysis, we manually isolated rotifers and transferred them to nutrient free COMBO media for one hour to allow for the emptying of gut.

Life History Experiment in LP Food

Alternative life history strategies may be favored in response to selection for fast growth in P-poor environments, thus we conducted a life history experiment in low-P food with the populations used in the second common garden (Table S2). Prior to the experiment individuals from batch cultures were isolated in 1 mL wells of tissue culture plates, provided with an LP-diet (1550 μmol L^{-1} C). The third generation was used for the experiment to minimize maternal effects (Zhou & Declerck 2020). During the life table 15-18 individuals from each population were monitored every two hours from birth until the production of the first juvenile or until confirmed as carrying a sexual egg.

Literature Cited

Declerck, S.A.J., Malo, A.R., Diehl, S., Waasdorp, D., Lemmen, K.D., Proios, K., *et al.* (2015). Rapid adaptation of herbivore consumers to nutrient limitation: eco-evolutionary feedbacks to population demography and resource control. *Ecol. Lett.*, 18, 553–562.

Kilham, S.S., Kreeger, D.A., Lynn, S.G., Goulden, C.E. & Herrera, L. (1998). COMBO: a defined freshwater culture medium for algae and zooplankton. *Hydrobiologia*, 377, 147–159.

Meirmans, P.G. & Van Tienderen, P.H. (2004). GENOTYPE and GENODIVE: two programs for the analysis of genetic diversity of asexual organisms. *Molecular Ecology Notes*, 4, 792–794.

Zhou, L., & Declerck, S.A.J. (2020). Maternal effects in zooplankton consumers are not only mediated by direct but also by indirect effects of phosphorus limitation. *Oikos*, 00, 1-9.
