# Supplementary material for: An experimental test of the growth rate hypothesis as a predictive framework for microevolutionary adaptation
Source: Ecology. 2022 Oct 23;104(1):e3853. doi: 10.1002/ecy.3853 (PMC10078216; doi:10.1002/ecy.3853)
Supplement: Supplementary file 3 — Appendix S3 [file ECY-104-0-s001.pdf]

Supporting information

Title: An experimental test of the growth rate hypothesis as a predictive framework for microevolutionary adaptation

Authors: Kimberley D. Lemmen, Libin Zhou, Spiros Papakostas, and Steven A.J. Declerck

Journal: Ecology

Appendix S3 Additional Data Analysis Information

Evolution Experiment

To explore temporal trends in population growth rate over the course of the evolution experiment for the two food quality treatments we fit both a piecewise and linear regression model. The model used for interpretation was chosen with the Akaike Information Criterion (i.e., with ΔAIC < approximately 2; Burnham & Anderson 2004). The piecewise regression was conducted using the package ‘segmented’ (Muggeo 2008), and the initial ‘breakpoint’ of the model was determined after a visual inspection of the plotted data. Davies test provided by the segmented package was applied to determine the significance of differences between slopes. Observations of population growth rate from all seven populations in each selection history were used for this analysis.

Trait Value Simulation of Neutrally Evolved Ancestral Populations and Comparison to Populations Evolved in the Evolution Experiment

For each trait in both common garden treatments we constructed a distribution of differences in mean trait values between a simulated ‘neutrally evolved’ ancestral population (i.e., change in phenotype due to drift associated with the repeated subsampling of small populations) and the

mean of values drawn from a distribution representing the populations at the end of the evolution experiment. For the neutral evolution simulations, we subjected virtual ancestral populations to exactly the same manipulations as those that were experienced by the real populations throughout the evolution and common garden experiments (see illustration below). Neutral evolution of ancestral populations was simulated and compared to the evolved populations 10,000 times according to the following steps:

1. Generate 30 seed genotypes by drawing from a normal distribution with the same mean and variance of the trait as measured for the subset of seed genotypes that were characterized in the common garden experiment.
2. Generate three replicate ancestral populations composed of two individuals of each of the 30 seed genotypes.
3. In each of these replicate populations, allow all genotypes to grow at identical rates, equal to what was observed in each of the food quality treatments (i.e., 0.85 and 0.267 day^{-1} in the HP and LP treatments, respectively). As in the experiment, at 24 hours intervals, 60 individuals are randomly selected to restart the populations. Changes in clonal frequencies within a replicate overtime (i.e., evolution) will thus only be due to chance events associated by the random selection of a subset of individuals during each transfer.
4. As in the real evolution experiments, allow populations to evolve during 36 time intervals.
5. After 36 time intervals, subsample each of the evolved populations in the same way as what was done to start technical replicates for populations as in the common garden experiment: for PGR and population structure randomly allocate 40 individuals from day

36 to four populations. For elemental traits we simulated no technical replicates to match the design of the second common garden experiment.

6. Identify the genotype of each individual in each population and assign its corresponding trait value. For each population, calculate the weighed mean trait value across genotypes with genotype weights corresponding to their relative abundance.
7. The trait value for the ancestral population is calculated as the mean of the three replicate populations ($MEAN_{neutr\_sim}$).
8. For each selection history (LP-selected, HP-selected and hybrid) draw three values from a normal distribution with the same mean and variance as observed for the population level traits measured for the corresponding selection history in the common garden experiment. Take the mean of these three traits ($MEAN_{obs}$).
9. Calculate the difference (ΔMEAN) between $MEAN_{neutr\_sim}$ and $MEAN_{obs}$.

The frequency distribution of 10,000 ΔMEAN-values was used to determine the probability that the observed trait change of the evolved populations differ from a neutrally evolved ancestral population. If 97.5% of the simulated differences were either all larger or smaller than zero (i.e., no difference between the observed and the neutrally evolved population), then the trait values observed in the evolved populations were considered significantly different from neutral expectations. P-values were calculated according to the following equation, where X is the simulated distribution:

$$2 * min\{P(X \leq 0)|P(X \geq 0)\}$$

Visual representation of the simulation for ancestral populations (next page). Subscripts S, A, and R stand for seed genotype, ancestral population replicate, and common garden pseudo replicate, respectively.

A. Trait values for 30 genotypes are generated from a normal distribution with same mean and variance as observed for the seed genotypes. Each circle represents a single genotype with its own trait value. Colored circles represent three of the genotypes drawn from the normal distribution.

B. Three replicate ancestral populations are each composed of two individuals per genotype.

C. Drift resulting from the daily random subsampling of the populations has affected the relative abundance of genotypes at the end of the evolution experiment

D. Forty individuals are randomly allocated (without replacement) to the four (pseudo)replicate populations of the common garden experiment. The trait value for the ancestral population is calculated as the mean of these four (pseudo)replicate populations.

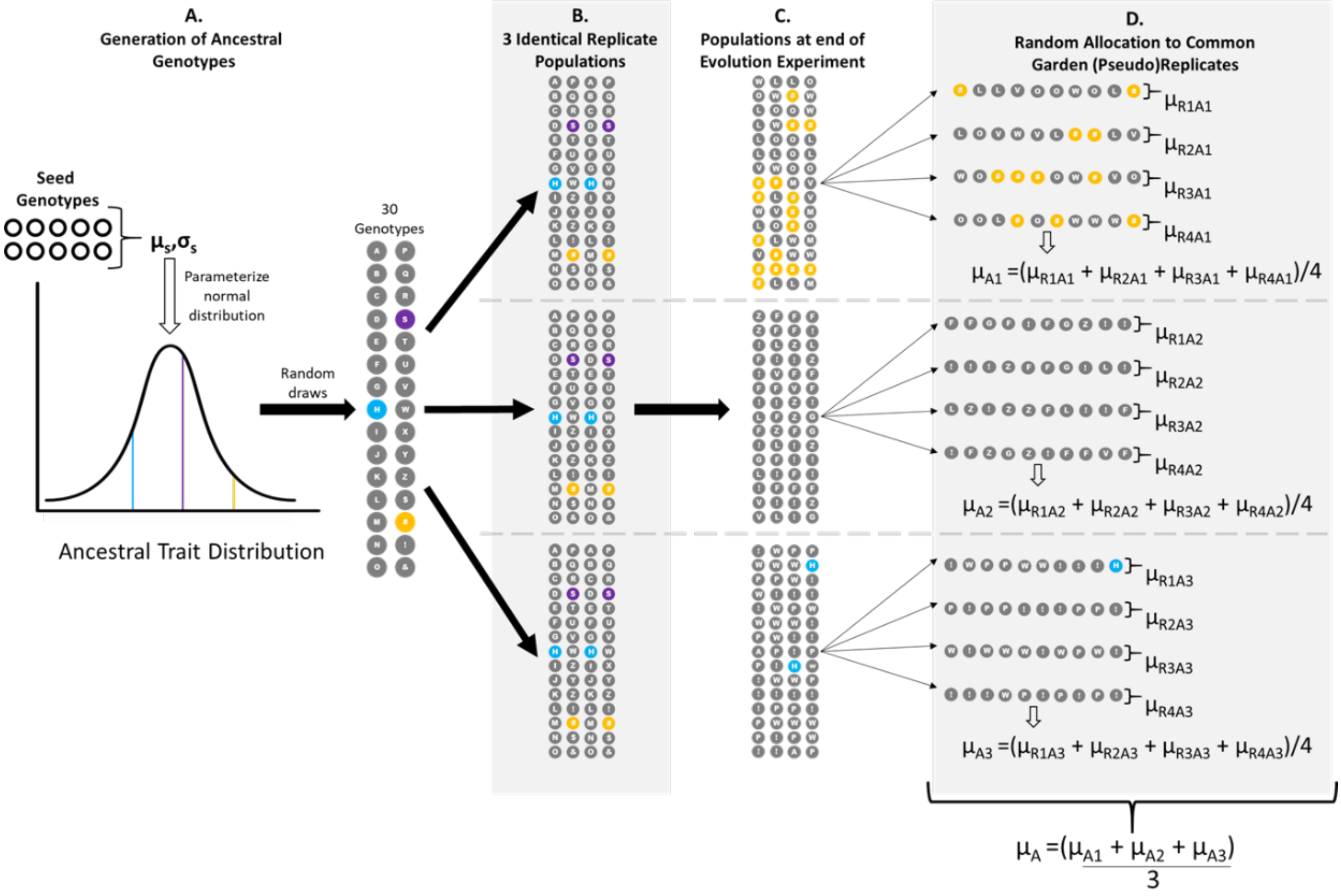
A.
Generation of Ancestral Genotypes
Seed Genotypes
μs,σs
Parameterize normal distribution
Ancestral Trait Distribution
Random draws
30 Genotypes
B.
3 Identical Replicate Populations
C.
Populations at end of Evolution Experiment
D.
Random Allocation to Common Garden (Pseudo)Replicates
μR1A1
μR2A1
μR3A1
μR4A1
μA1 =(μR1A1 + μR2A1 + μR3A1 + μR4A1)/4
μR1A2
μR2A2
μR3A2
μR4A2
μA2 =(μR1A2 + μR2A2 + μR3A2 + μR4A2)/4
μR1A3
μR2A3
μR3A3
μR4A3
μA3 =(μR1A3 + μR2A3 + μR3A3 + μR4A3)/4
μA =(μA1 + μA2 + μA3)/3

Literature Cited

Burnham, K.P. & Anderson, D.R. (2004). Multimodel inference: understanding AIC and BIC in model selection. *Sociol. Meth. Res.*, 33, 261–304.

Muggeo, V.M.R. (2008). segmented: an R Package to Fit Regression Models with Broken-Line Relationships. *R News*, 8/1, 20-25.
