# Supplementary material for: An experimental test of the growth rate hypothesis as a predictive framework for microevolutionary adaptation
Source: Ecology. 2022 Oct 23;104(1):e3853. doi: 10.1002/ecy.3853 (PMC10078216; doi:10.1002/ecy.3853)

Supporting information

Title: An experimental test of the growth rate hypothesis as a predictive framework for microevolutionary adaptation

Authors: Kimberley D. Lemmen, Libin Zhou, Spiros Papakostas, and Steven A.J. Declerck

Journal: Ecology

Appendix S4 Supplementary Figures

Figure S1. Temporal trends in growth rate of populations during the evolution experiment. A) HPF selection treatment, the blue line represents a linear response of population growth without breakpoints (see Table S3). B) LPF selection treatment the red line represents the two segments of a segmented regression analysis with a breakpoint at 13.5 days. Segment point and 95% confidence interval is represented by the upper grey symbol and horizontal error bar.

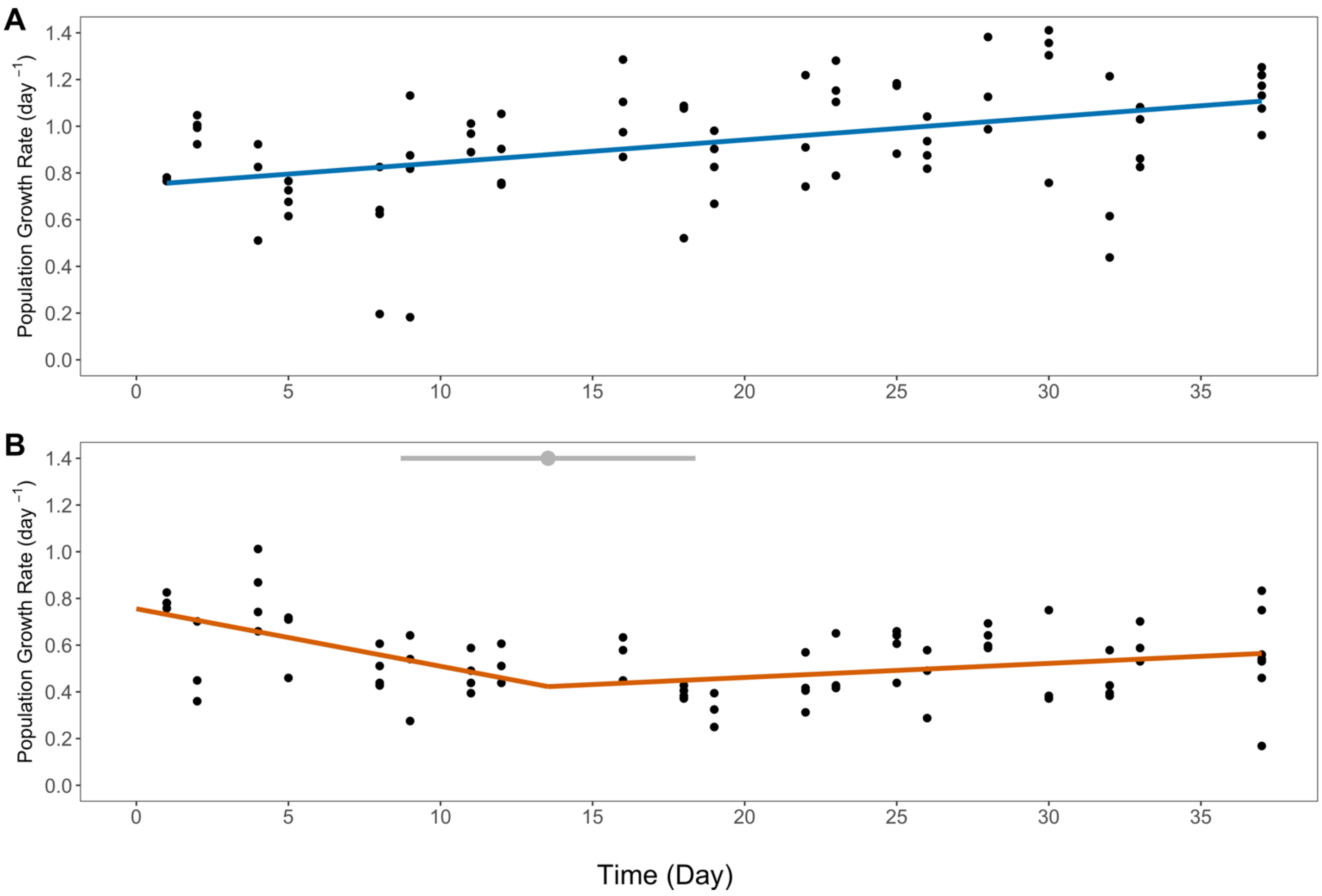

Figure S2. Number of resting eggs observed during the evolution experiment in populations of the high (HFP) and low (LPF) phosphorus treatments, respectively. Symbols represent means across populations (n=7) and error bars represent ± 2 standard errors.

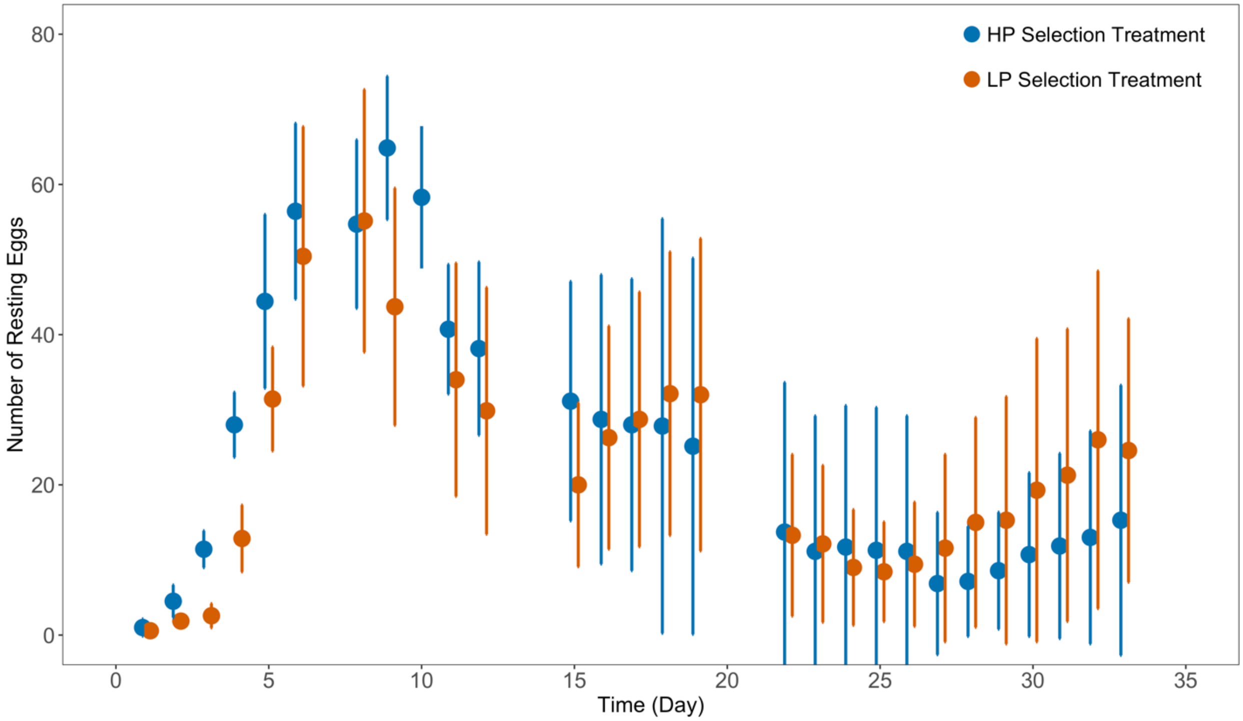

Figure S3. Fraction of sexual individuals in the non-hybrid and ancestral populations in the first common garden with high (HPF) and low phosphorus diets (LPF). Non-hybrid populations were selected in either high (HP) or low phosphorus (LP) selection regimes. The fraction of sexual females was calculated as the number of females with sexual eggs (male and diapausing eggs) divided by the total number of mature individuals (i.e., adults with male, diapausing, or amictic eggs). For non-hybrid populations we present means ± 2 standard errors (solid line; n=3). For the ancestral population, means and 95% confidence intervals were obtained by bootstrapping the values observed for a subset of seed genotypes (dashed line; Table S10).

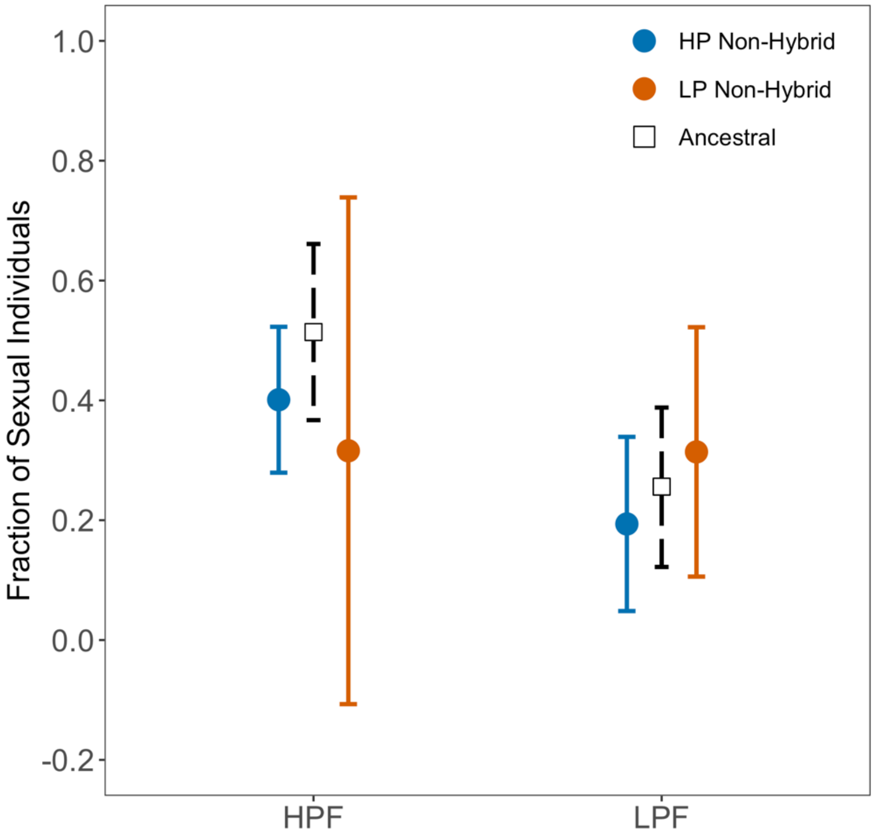

Figure S4. Response of individual N content, and molar N:C and N:P ratios of non-hybrid and hybrid populations in the second common garden experiment when exposed to high (HPF) and low phosphorus diets (LPF). During the evolution experiment, non-hybrid populations were selected in either high (HP, n=3) or low phosphorus (LP, n=3) treatments. Hybrid populations from HP and LP selection regimes are represented separately (n=1). For non-hybrid and hybrid populations we present means ± 2 standard errors (solid line). For the ancestral population means and 95% confidence intervals were obtained by bootstrapping the values observed for a subset of seed genotypes (dashed line; Table S10).

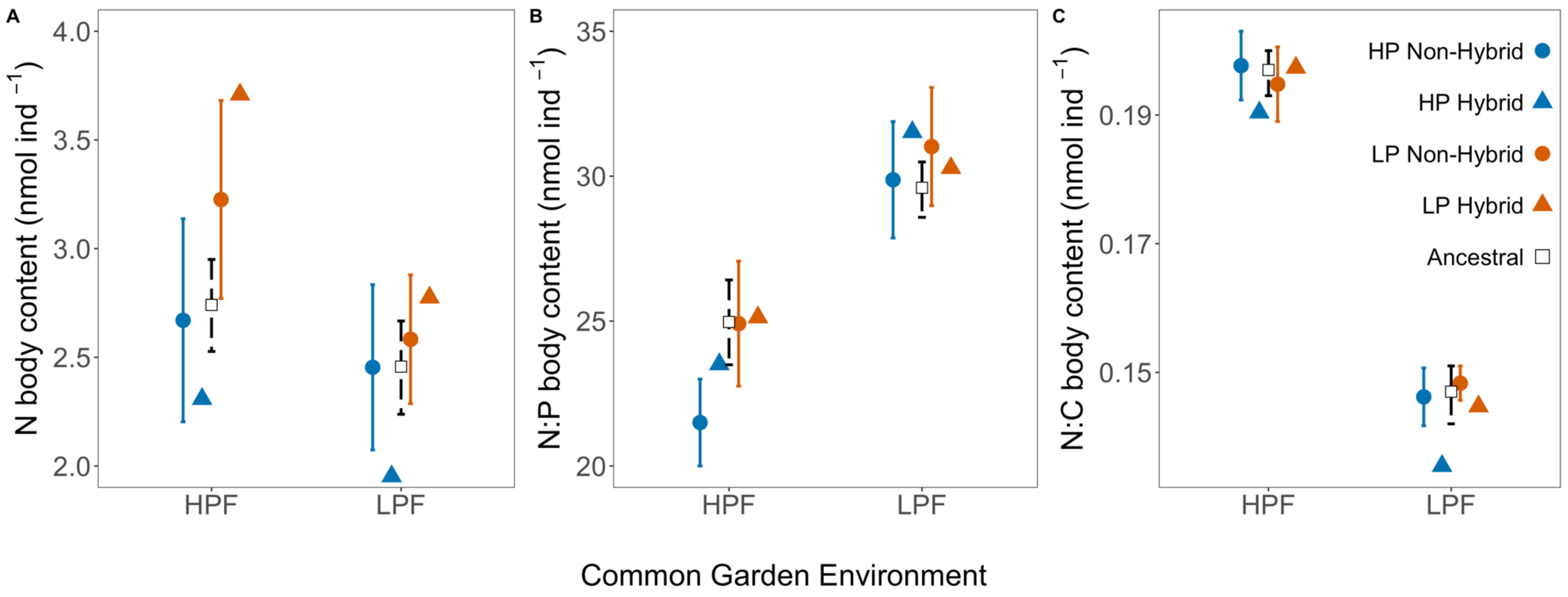

Figure S5. Comparison of traits between non-hybrid and hybrid populations. Populations are compared in the two food quality treatments of the common garden experiments corresponding to their selection history in the evolution experiment (i.e., LP and HP-selected populations in LPF and HPF treatments, respectively). The fraction of sexual females was calculated as the number of females with sexual eggs (male and diapausing eggs) divided by the total number of mature individuals (i.e., adults with male, diapausing, or amictic eggs). Non-hybrid populations are represented according to their selection history in the evolution experiment. The response of hybrid populations from HP and LP selection regimes were combined for analysis (See Figure 1, Table 1). For evolved populations we present means ± 2 standard errors of observed vales (non-hybrid, n=3; hybrid, n=2). For the ancestral population means and 95% confidence intervals were obtained by bootstrapping the values observed for a subset of seed genotypes (dashed line; Table S10).

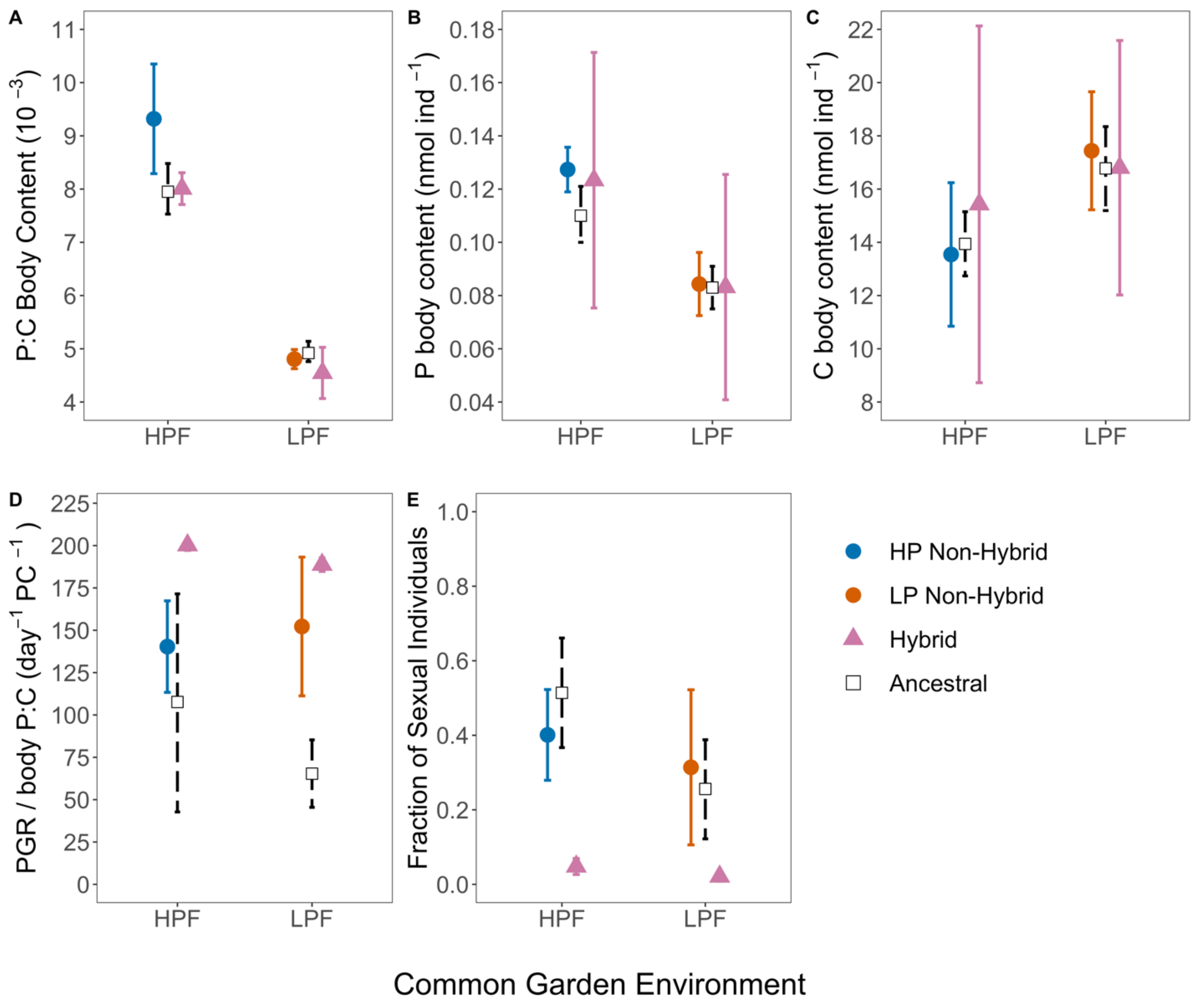

Supplement: Supplementary file 4 — Appendix S4 [file ECY-104-0-s002.pdf]
